# Nutritional control regulates symbiont proliferation and life history in coral-dinoflagellate symbiosis

**DOI:** 10.1186/s12915-022-01306-2

**Published:** 2022-05-13

**Authors:** Guoxin Cui, Yi Jin Liew, Migle K. Konciute, Ye Zhan, Shiou-Han Hung, Jana Thistle, Lucia Gastoldi, Sebastian Schmidt-Roach, Job Dekker, Manuel Aranda

**Affiliations:** 1 Biological and Environmental Sciences and Engineering Division (BESE), Red Sea Research Center (RSRC), King Abdullah University of Science and Technology (KAUST), Thuwal, Saudi Arabia; 2 Program in Systems Biology, Department of Biochemistry and Molecular Pharmacology, University of Massachusetts Medical School, Worcester, MA USA; 3 Howard Hughes Medical Institute, Chevy Chase, MD USA

**Keywords:** Coral-Symbiodiniaceae symbiosis, Symbiont proliferation, Life history, Symbiont population control, Coral, Symbiodiniaceae, Photosymbiosis

## Abstract

**Background:**

The coral-Symbiodiniaceae symbiosis is fundamental for the coral reef ecosystem. Corals provide various inorganic nutrients to their algal symbionts in exchange for the photosynthates to meet their metabolic demands. When becoming symbionts, Symbiodiniaceae cells show a reduced proliferation rate and a different life history. While it is generally believed that the animal hosts play critical roles in regulating these processes, far less is known about the molecular underpinnings that allow the corals to induce the changes in their symbionts.

**Results:**

We tested symbiont cell proliferation and life stage changes in vitro in response to different nutrient-limiting conditions to determine the key nutrients and to compare the respective symbiont transcriptomic profiles to cells *in hospite*. We then examined the effects of nutrient repletion on symbiont proliferation in coral hosts and quantified life stage transitions in vitro using time-lapse confocal imaging. Here, we show that symbionts *in hospite* share gene expression and pathway activation profiles with free-living cells under nitrogen-limited conditions, strongly suggesting that symbiont proliferation in symbiosis is limited by nitrogen availability.

**Conclusions:**

We demonstrate that nitrogen limitation not only suppresses cell proliferation but also life stage transition to maintain symbionts in the immobile coccoid stage. Nutrient repletion experiments in corals further confirmed that nitrogen availability is the major factor limiting symbiont density *in hospite*. Our study emphasizes the importance of nitrogen in coral-algae interactions and, more importantly, sheds light on the crucial role of nitrogen in symbiont life history regulation.

**Supplementary Information:**

The online version contains supplementary material available at 10.1186/s12915-022-01306-2.

## Background

The symbiotic relationship between corals and Symbiodiniaceae is the cornerstone underlying the success of corals in the oligotrophic environment of tropical seas. In this partnership, the host provides inorganic nutrients to the symbionts, while the symbionts translocate photosynthesis-fixed organic carbons to the host, covering 80–100% of its energetic requirements [1–3]. Despite the importance of this association, the mechanisms underlying symbiont control are still poorly understood [4]. Most essential are the questions of how the host regulates symbiont life history and proliferation.

Like other dinoflagellates, Symbiodiniaceae have two major life stages: a flagellated, motile mastigote stage, and a non-motile coccoid stage [5]. In culture, cell division is exclusive to the coccoid stage and results in the emergence of two motile mastigote cells that actively swim until they shed their flagella towards the end of the photoperiod and metamorphose into the coccoid form [5]. However, once *in hospite*, this life history is dramatically changed and no motile mastigotes are observed, i.e., all symbiotic cells are exclusively coccoid *in hospite* [5–7]. While it is generally assumed that the host animals play critical roles in regulating this change, little is known about the underlying mechanisms.

Aside from the life history change (mastigote/coccoid), another prominent feature for the symbionts *in hospite* is the severe reduction in proliferation rates (cell divisions). Comparatively, the latter has been more extensively examined, and often linked to the availability of various nutrients that are potentially regulated by host cells. Among all the nutrients, nitrogen, phosphate, iron, and other trace metals have been suggested as potential key factors that could allow the host to control Symbiodiniaceae [8–16]. Initial evidence came in the form of physiological experiments demonstrating that the growth of free-living Symbiodiniaceae largely relied on the availabilities of these nutrients [17–20]. Stoichiometry analyses show that algal symbionts from various cnidarian hosts are either lacking of nitrogen or devoid of phosphate [21–24]. Symbiont density fluctuates significantly according to the availabilities of these nutrients [25, 26]. In this context, it is interesting to note that nutrient uptake in freshly isolated symbionts is very low [27–30], in contrast to the high assimilation capacity of their symbiotic hosts [31–36]. This finding might point towards a potential competition between host and symbionts over these essential nutrients, which may underlie the control of symbiont proliferation *in hospite*. Such a relationship that is based on competition between both partners, and which could be described as reciprocal parasitism [37], would result in a highly efficient system for the recycling and assimilation of valuable nutrients that allows both partners to thrive in oligotrophic environments [38]. Recent transcriptomics and metabolomics studies have provided further support for the importance of these nutrients in host-symbiont interactions [8, 24, 39–41]. While the majority of these studies suggest that the cells *in hospite* are either nitrogen- or phosphate-limited, increasing evidence highlights the importance of iron and other trace metals in algal cell proliferation. However, as most of the studies investigated the nutrients in a singular manner, it is difficult to directly compare the effects of different nutrients on cell growth. Hence, a systematic evaluation that tested the effects of different nutrient components in parallel could gain a better understanding of algal symbiont growth regulation.

As the life stage transition is part of the proliferation process for free-living cells, it is possible that the factors affecting proliferation may directly regulate the cell life history. Accordingly, we hypothesize that nutrient limitation may play dual roles in suppressing cell proliferation and regulating symbiont life history. To examine the hypothesis, we tracked the proliferation rates and life stages of *Symbiodinium microadriaticum* under different nutrient-limited conditions in culture (nitrogen, phosphate, and iron, respectively) and compared transcriptional responses to *in hospite* conditions within the native coral host *Stylophora pistillata* (Additional file 1: Fig. S1). We found that nitrogen limitation is not only vital for symbiont population control but that it also plays a critical role in symbiont life history regulation. Our study further emphasizes the importance of balancing nitrogen metabolism between coral hosts and their algal symbionts [8, 42].

## Results

### Octocoral symbiont-binding lectin is absent in symbiotic anthozoans

Unlike the extensive research focused on symbiont proliferation control, little attention has been paid to the complex life history of Symbiodiniaceae and its regulation *in hospite*. A previous study addressing this issue proposed that a d-galactose binding lectin SLL-2 from octocoral *Sinularia lochmodes* could be involved in the processes of confining the dinoflagellate symbionts to the non-motile stage in octocorals [43]. To look for potential homologs of SLL-2 in other cnidarians, we analyzed the genomes of 20 symbiotic cnidarian species (Additional file 2: Table S1) available on the public marine genomics data repository [44]. However, we were not able to identify a single SLL-2 homolog in any of these species. This finding suggests that other mechanisms must exist in these symbiotic cnidarians. Considering the connections between algal life history and cell proliferation, we then decided to examine the potential effects of different nutrients on both processes.

### Setting up nutrient-depleted cultures of *S. microadriaticum*

Symbiodiniaceae cells take up and assimilate different nutrients at different rates and capacities [45]. For instance, Symbiodiniaceae can store nitrogen in the form of uric acid crystals and mobilize them when nitrogen availability drops [46, 47]. Similarly, surplus iron in the medium, especially during the early culturing stages when cell concentrations are low, could stick to the algal cell surface and make it difficult to adjust the functional concentration during the entire culturing phases [48]. Therefore, we started with identifying the nutrients that affect Symbiodiniaceae proliferation rates in culture and determining the levels of these nutrients that lead to the reduction of cell densities to half of the control conditions.

The f/2 medium used in culturing free-living Symbiodiniaceae consists of six components: nitrogen, phosphate, iron, other trace metals, EDTA, and vitamins (Additional file 2: Table S2). We performed an initial pilot experiment to test the relative importance of these components on cell proliferation by outright removing each of the six nutrient groups (while keeping the other five components at the original concentrations). We observed that nitrogen, phosphorus, and iron depletion led to an obvious reduction in proliferation rates in the cultures; on the other hand, the depletion of other non-iron trace metals, EDTA, and vitamins did not have an observable effect on culture growth rates after 6 weeks (data not shown).

**Fig. 1:**
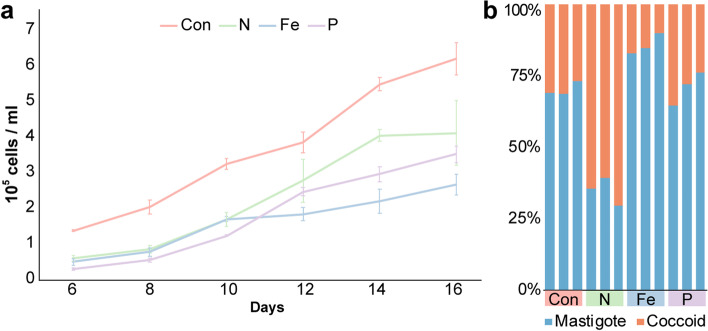
Growth of *S. microadriaticum*. **a** Cell proliferation curve of *S. microadriaticum* for days 6 to 16 after subculturing in different free-living conditions. Error bars represent standard errors of the mean. *n* = 3. **b** Percentage of mastigote and coccoid in *S. microadriaticum* grown in different free-living conditions. Con, control condition in unmodified f/2 medium; N, nitrogen-limited condition that one-quarter of the original nitrate concentration was used; Fe, iron-limited condition that iron concentration reduced to a hundredth of its original in f/2; P, phosphate-limited condition that phosphate was at an eighth of its original concentration in f/2. Each bar stands for a biological replicate

Based on these observations, we set up long-term (2 years) nitrogen-, phosphate-, and iron-depleted cultures, along with f/2 controls (Additional file 2: Table S3). In these cultures, nutrients were selectively depleted to produce cell densities that were approximately half of the control cultures in full f/2 medium at late exponential to stationary phase ( ~3 × 10^5^ vs ~ 6 × 10^5^ cells/ml at day 16 post-inoculation; Fig. 1a). These corresponded to 1/4 of the original nitrate concentration in f/2 (labeled as “N”), 1/8 of the original phosphate concentration (“P”), or 1/100 of the original iron concentration (“Fe”).

### Effect of nutrient depletion on symbiont life history

To examine the effects of nutrient limitation on symbiont life history, we separately assessed the number of mastigote and coccoid cells under nutrient-depleted conditions (Fig. 1b). The mastigote/coccoid ratios were similar between control and phosphate-limited samples (mean ratio ± s.e.m., 2.39 ± 0.17 vs 2.54 ± 0.39, paired *t*-test *p* = 0.74). Iron limitation increased the proportion of mastigote cells in comparison with control (mean percentage ± s.e.m., 85.75% ± 2.14% vs 70.36% ± 1.40%, paired *t*-test *p* = 0.004). Coccoid cells became dominant (65.20% ± 2.92%) only in the nitrogen-limited condition (N vs Con, paired *t*-test *p* = 0.0004). This indicated that the limitation of nitrogen significantly increased the proportion of non-motile cells of free-living cells similar to *in hospite* conditions.

Combining the growth curves with the coccoid/mastigote ratios, we asked if the change in mastigote/coccoid ratios could be a side effect of the cell proliferation suspension caused by nutrient limitations. To remove this potential bias and evaluate the direct effect of nutrient limitation on cell morphology, we performed Fisher’s exact tests on coccoid and mastigote cell numbers. Based on these tests, phosphate limitation did not affect the life stage (Fisher’s exact *p* = 0.34); iron depletion promoted the motile mastigote stage (Fisher’s exact *p* = 6.89e−26), while reducing nitrogen significantly increased the ratio of coccoid cells (Fisher’s exact *p* = 2.93e−21). This revealed that limiting nitrogen availability indeed confines *S. microadriaticum* cells to the coccoid stage under free-living conditions.

### Effect of nutrient depletion on symbiont gene expression profiles

**Fig. 2:**
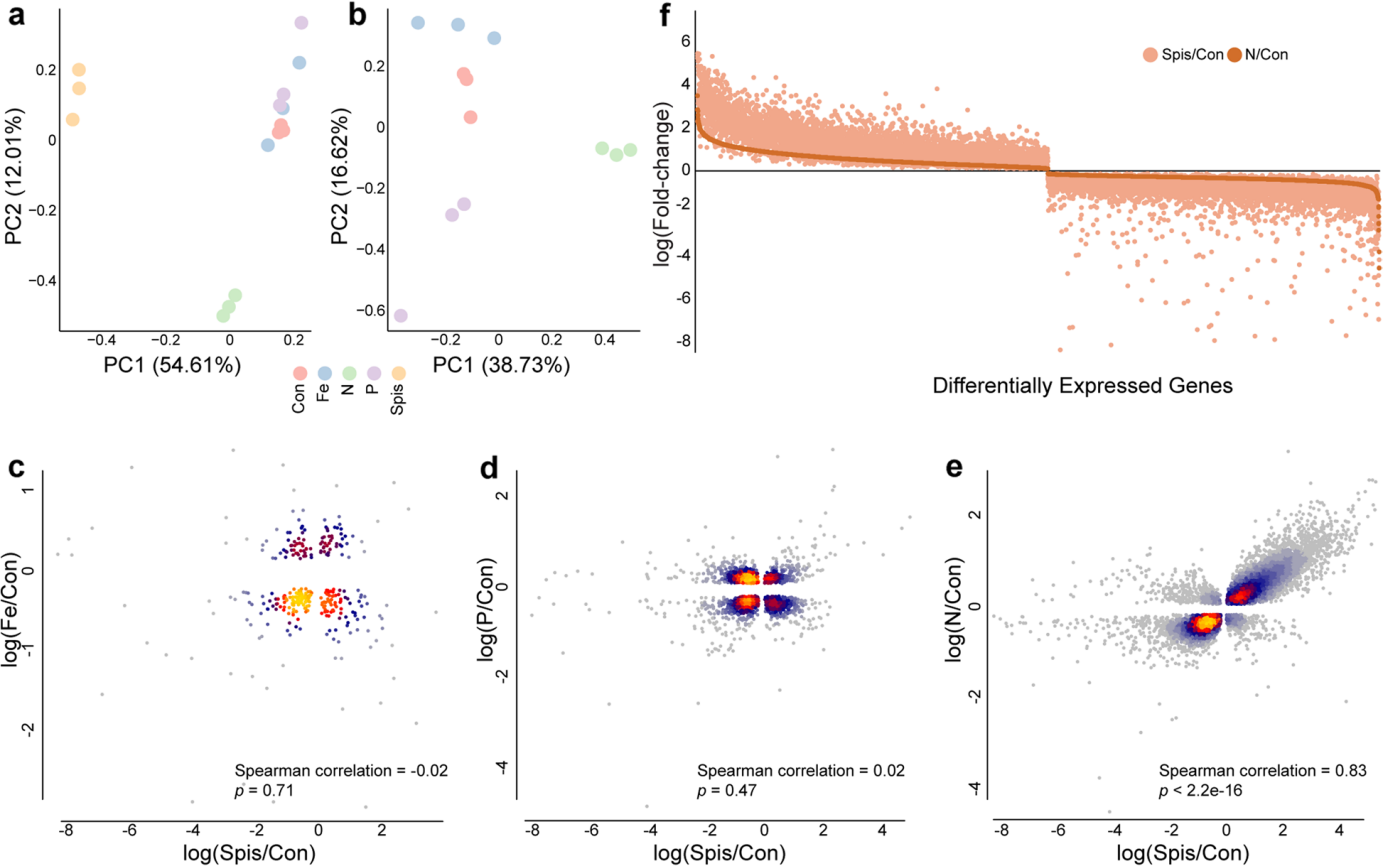
Gene expression profiling of *S. microadriaticum*. **a** Principal component analysis of transcriptomic profiles of algal cells living *in hospite* (Spis) and *ex hospite* (Con, Fe, N, P). **b** Principal component analysis of transcriptomic profiles of algal cells living in different culturing conditions: Control, iron (Fe), nitrogen (N), and phosphate limitation (P). **c** Spearman correlation of expression changes between iron-limited and *in hospite S. microadriaticum*, in comparison with control cells. Each dot represents a differentially expressed gene identified from both conditions in comparison with *ex hospite* control. The correlation coefficient and *p*-value were calculated based on their log-transformed fold changes. Colors represent the dot density which corresponds to increasing density from gray, blue, and red to yellow. **d** Spearman correlation of expression changes between phosphate-limited and *in hospite S. microadriaticum*, in comparison with control cells. **e** Spearman correlation of expression changes between nitrogen-limited and *in hospite S. microadriaticum*, in comparison with control cells. **f** Expression changes of co-directionally changed genes in Spis/Con and N/Con. The genes on the *x*-axis were ranked by log-transformed fold changes in N/Con comparison. Colors represent the two comparisons as indicated: dark orange is for N/Con, while light orange is for Spis/Con

We then asked whether nitrogen limitation correlated with the observed reduction in growth and changes in life stage and whether the mechanism is shared with symbionts *in hospite*. To answer the question, using RNA-seq, we quantified the expression level of 49,109 gene models of *S. microadriaticum* from 12 long-term cultures (*n* = 3 from 4 conditions: nitrogen-, phosphate-, and iron-limited cultures; and f/2 controls). We also mapped publicly available RNA-seq data from *S. pistillata* (the natural host of *S. microadriaticum*; *n* = 3) against the same *S. microadriaticum* gene models to obtain *in hospite* expression profiles of *S. microadriaticum*. All samples had over 10 million mapped reads which are sufficient to profile the transcriptomic responses of *S. microadriaticum* in the different conditions. Based on the principal component analysis, cells from different conditions exhibited distinct expression profiles (Fig. 2a, b). While free-living cells clustered better with each other than with cells *in hospite* (Fig. 2a), the expression of nitrogen-limited free-living cells were divergent from the other cultured samples (Fig. 2b).

In order to understand the transcriptomic changes across the cultures, we performed four differential gene expression comparisons using f/2 controls as a baseline: symbionts *in hospite* (Spis/Con, Additional file 3: Table S4), nitrogen limitation (N/Con, Additional file 4: Table S5), phosphate limitation (P/Con, Additional file 5: Table S6), and iron limitation (Fe/Con, Additional file 6: Table S7). We found starkly different numbers of differentially expressed genes (DEGs) identified in each analysis (Additional file 1: Fig. S2, Additional files 3, 4, 5 and 6: Table S4-S7). Interestingly, symbiosis with the animal host caused the largest number of genes to be differentially expressed in *S. microadriaticum*, followed by nitrogen-, phosphate-, and iron-limited treatments.

### Gene expression profiles of *S. microadriaticum* in different conditions

To gain insight into the nutritional status of symbionts *in hospite*, we compared the overall gene expression changes of *S. microadriaticum* in response to symbiosis and nutrient-limited culturing conditions (Additional file 1: Fig. S3). All three comparisons showed extreme statistical significance (*p* < 2.2e−16). However, a positive correlation (Spearman’s *ρ* = 0.63) was only identified between nitrogen-limited cells and symbionts *in hospite*, whereas no correlation was shown for iron- and phosphate-limited cells. Although we observed such notable differences here, we were curious if higher similarities would show up in the overlap of DEGs. Further testing of the correlations between DEGs identified from the four differential expression analyses confirmed that the sole significant correlation was between nitrogen-limited and *in hospite* conditions (Fig. 2c–e).

Although we found significant intersections of DEGs (Fisher’s exact test, *p* < 2.2e−16) between *in hospite* and nitrogen-limited cells (12,295 DEGs in common), phosphate-limited cells (2037 DEGs in common), or iron-limited cells (329 DEGs in common), the directionalities of their expression changes varied with condition (Additional files 3, 4, 5 and 6: Table S4-S7). However, it is interesting to note the highly significant co-directionality (binomial test: *p* < 2.2e−16) of the expression changes observed for nitrogen-limited cultures and *in hospite* symbionts, with 92.67% of the 12,295 (11,394) DEGs changing expression in the same direction (Fig. 2e). In contrast, there was no evidence for similar co-directional expression changes of Spis/Con against Fe/Con (54.10% co-directionally changed, binomial test: *p* = 0.08; Fig. 2a) or against P/Con (47.08% co-directionally changed, binomial test: *p* = 0.996; Fig. 2b). More interestingly, we found that 92.03% of the co-directionally expressed DEGs (10,486) showed a higher magnitude of change under *in hospite* conditions (Fig. 2f).

### Pathway activity changes in response to different nutrient conditions

**Fig. 3:**
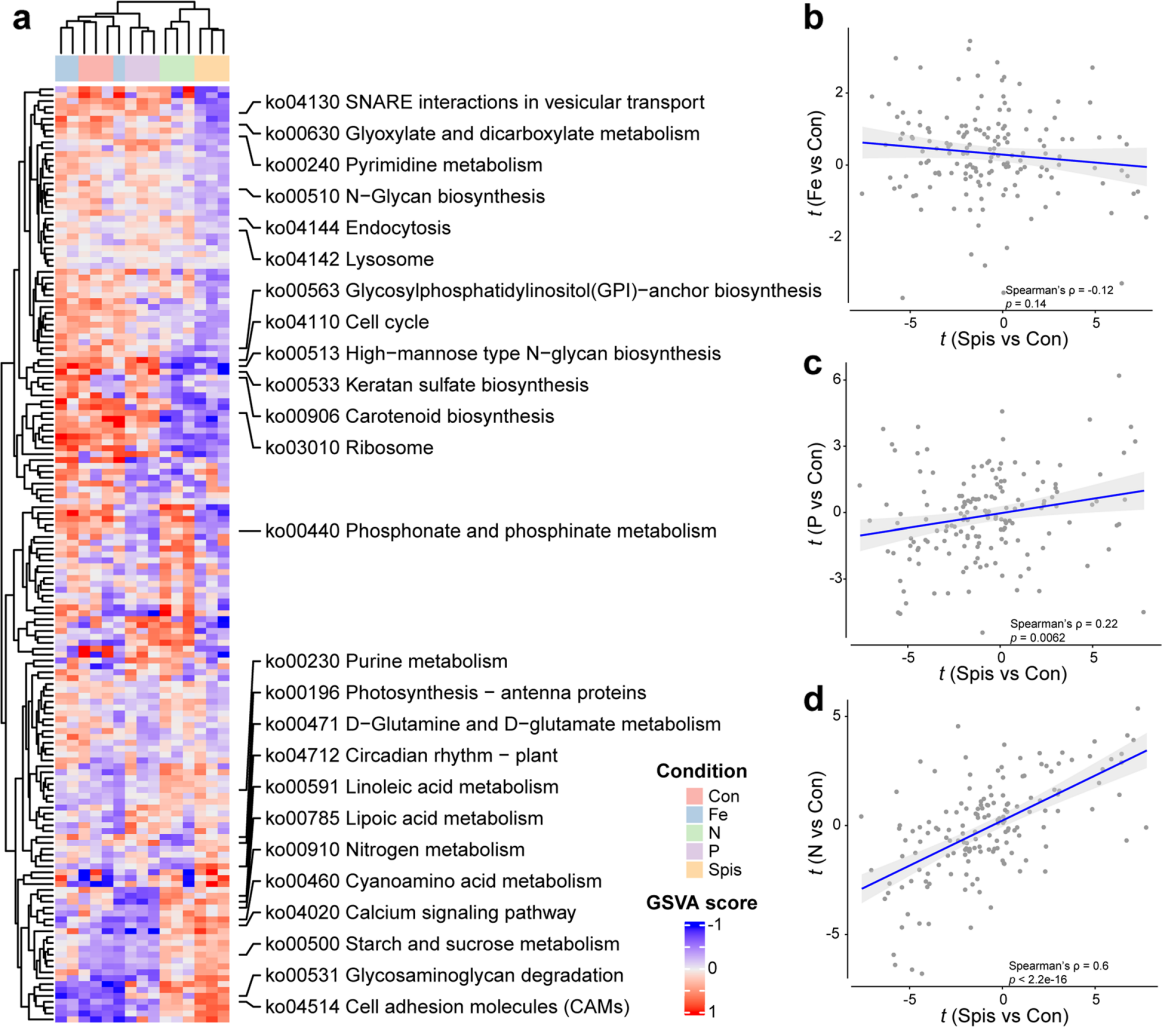
Pathway activity changes of *S. microadriaticum* in response to different growing conditions. **a** Heatmap of GSVA scores representing the pathway activities calculated based on the expression levels of genes associated with each pathway. The dendrogram on top indicates the hierarchical clustering of samples at the pathway level. Scatter plots demonstrate the correlations of pathway activity changes, denoted by *t* values, between Spis/Con and Fe/Con (**b**), P/Con (**c**), or N/Con (**d**). The blue line and gray area represent the linear regression line and its 95% confidence interval, respectively

To further interpret the gene expression changes at systemic levels, we estimated the activities of genome-annotated pathways of cells under the different nutrient conditions. Consistent with our gene-level analysis, nitrogen-limited *ex hospite* cells clustered better with cells *in hospite* at the pathway level, compared to control and iron- and phosphate-limited samples (Fig. 3a, Additional file 7: Table S8). We quantified the activity changes of these pathways by contrasting their activity scores of Spis, N, Fe, and P with Con using a generalized linear model implemented in limma [49]. *t* values were obtained to represent the pathway activity changes as previously suggested [50]. Further correlation analyses indicated that no correlation was observed between the *t* values of iron-limited and *in hospite* cells (Fig. 3b), and a moderate correlation was found between phosphate limitation and *in hospite* condition (Fig. 3c), while nitrogen-limited cells showed the highest correlation with symbiont *in hospite* (Fig. 3d).

Moreover, we found that *S. microadriaticum* appeared to activate or deactivate certain sets of pathways in response to the change in culturing conditions and/or symbiotic state (Fig. 3a, Additional file 7: Table S8). Pathways associated with cell proliferation (ko04110 Cell cycle, ko03010 Ribosome, etc.) were suppressed in both nitrogen-limited *ex hospite* and *in hospite* cells. On the other hand, pathways involved in nitrogen metabolism (ko00910 nitrogen metabolism, ko00471 d-glutamine, and d-glutamate metabolism, etc.) were generally induced in cells from these two conditions. Based on the systemic pathway-level analysis, we were able to identify many pathways altered by nutrient availability of algal cells that were obscured in our initial gene-level study. The processes associated with uptake, transport, and metabolizing of exogenous nutrients—ko04144 endocytosis, ko04130 SNARE interactions in vesicular transport, and ko04142 Lysosome—were specifically inhibited in cells *in hospite*. Most importantly, we found that many nitrogen-saving strategies were employed either solely in cells *in hospite* or shared between symbiotic cells and nitrogen-limited *ex hospite* cells. Ammonium-producing biological processes, such as ko00230 Purine metabolism, ko00460 cyanoamino acid metabolism, and ko00531 glycosaminoglycan degradation, were activated, while the activities of nitrogen-consuming pathways, pyrimidine metabolism (ko00240) and *N*-glycan biosynthesis (ko00510, ko00513, and ko00533), were generally decreased (Fig. 3a, Additional file 7: Table S8).

In addition, we found that both antenna proteins and plant circadian rhythm pathways were highly upregulated in symbiotic cells, which was distinct from nitrogen-limited *ex hospite* cells. Unlike photosynthesis-related pathways, the processes potentially determining mastigote-coccoid transition showed similar patterns between nitrogen-limited *ex hospite* and *in hospite* cells. GPI anchor proteins and cell adhesion molecules play important roles in cell membrane organization, cell wall synthesis, and cell adhesion. Activation of the associated pathways (ko00563 and ko04514) by nitrogen limitation in both symbiotic and free-living cells indicates nitrogen limitation-mediated cell life stage regulations might be a shared mechanism between cells in culture and *in hospite* (Fig. 3a, Additional file 7: Table S8).

### Nitrogen-depletion induces life history changes

**Fig. 4:**
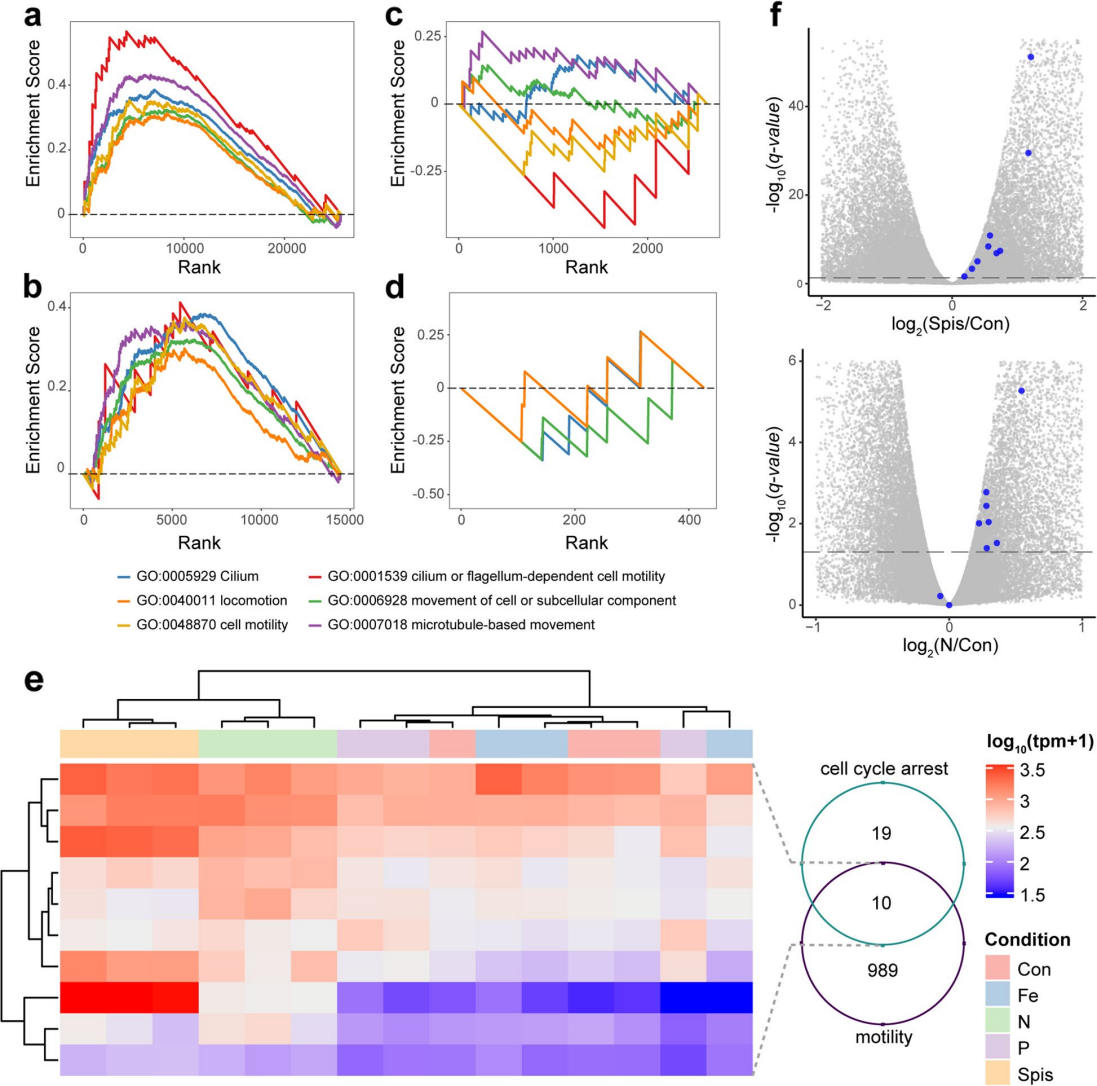
Nitrogen limitation regulates algal life history by inducing expression changes of genes associated with motility and cell cycle progression. Enrichment of cell motility-related gene sets in DEGs identified from Spis vs Con (**a**), N vs Con (**b**), P vs Con (**c**), and Fe vs Con (**d**). The *y*-axis represents running enrichment scores calculated by GSEA, while the *x*-axis shows the rank of associated genes in DEGs identified from each of the differential expression comparisons. The increment levels of the enrichment scores indicate the correlation of the corresponding gene set with the phenotypic change of the cells under different treatments. **e** Expression level of genes involved in both cell motility and cell cycle arrest under different conditions. Venn diagram indicates the overlapped 10 genes involved in both cell cycle arrest and cell motility. **f** Volcano plots indicate the expression changes of polycystin-2 genes in Spis and N, compared to free-living control condition. Blue dots represent the nine polycystin-2 genes in *S. microadriaticum*

To further investigate the mechanism underlying the life stage regulation, we performed gene set enrichment analyses (GSEA) on multiple cell motility- and flagellum-related GO terms, since the most striking difference between mastigotes and coccoid cells is the presence of flagella and flagella-associated motility. We found that not only DEGs identified from Spis vs Con (Fig. 4a) and N vs Con (Fig. 4b) were significantly enriched with these GO terms but also their enrichment levels were surprisingly similar. In contrast to this, there was no such enrichment pattern for the DEGs identified from P vs Con (Fig. 4c) and Fe vs Con (Fig. 4d).

We then examined if nitrogen limitation-induced cell motility changes are linked with cell proliferation reduction by looking into the genes associated with both of these GO terms and KEGG pathways. We found ten genes involved in both processes, of which nine encoded polycystin-2 proteins. Symbionts *in hospite* shared similar expression levels of these ten genes with nitrogen-limited cells, but they showed distinct patterns in the other conditions (Fig. 4e). Differential expression analysis indicated that all nine polycystin-2 genes were upregulated in cells *in hospite*, while seven showed the same change under nitrogen-limited conditions (Fig. 4f). This indicated that polycystin-2 might play crucial roles in algal cells in response to nitrogen limitation.

### Effects of nitrogen availability on *ex hospite* and *in hospiteS. microadriaticum*

**Fig. 5:**
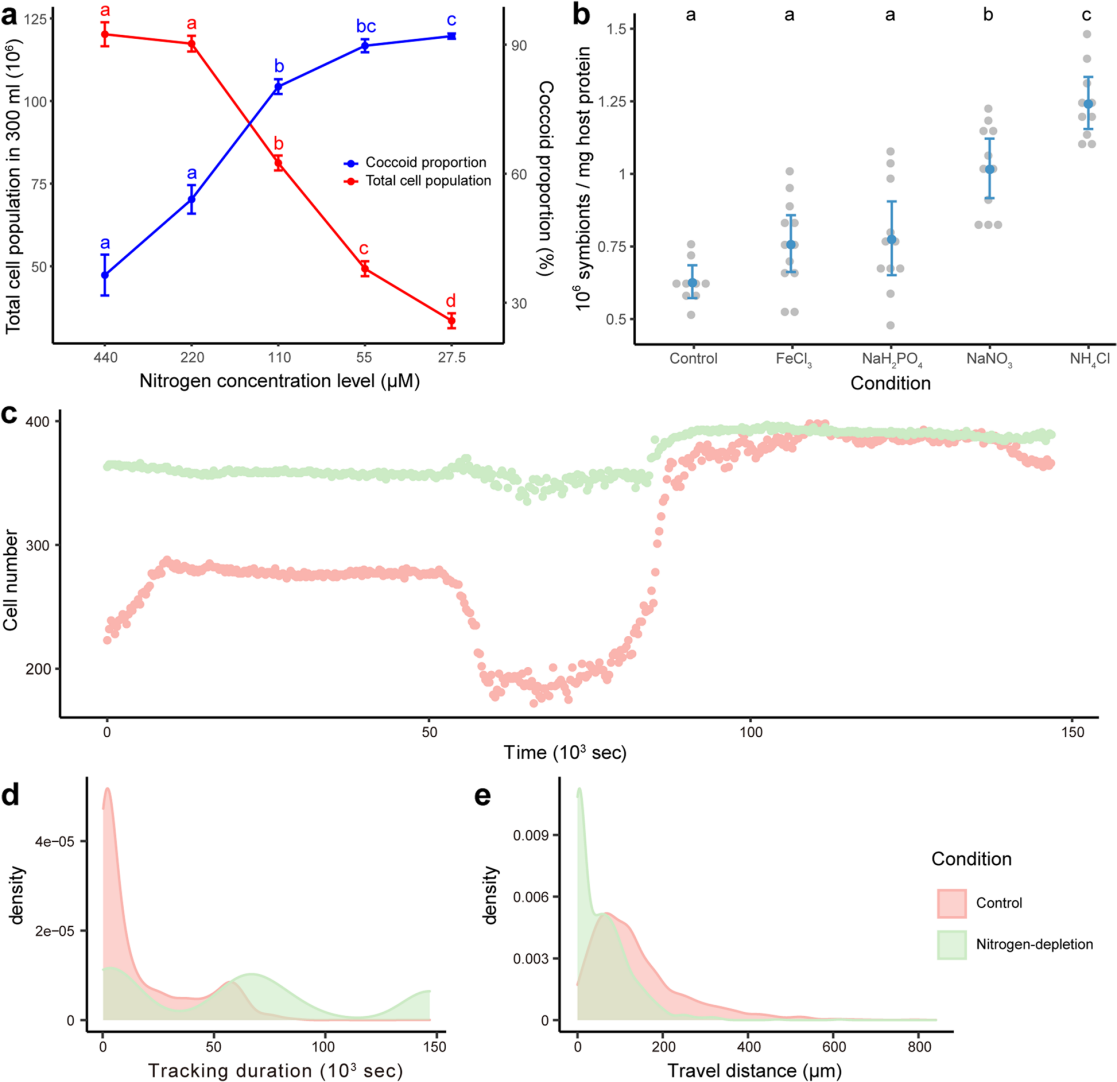
Effects of nitrogen availability on *S. microadriaticum*. **a** Cell populations and coccoid proportions of *S. microadriaticum* in different nitrogen-limited conditions. **b** Symbiont cell density of coral fragments supplied with different nutrients. In both figures, colored dots and error bars represent the mean ± 95% confidence intervals of symbiont cell density. Letters indicate the statistical differences between treatments which were calculated using one-way ANOVA with Games-Howell post hoc tests. **c** Real-time cell number changes over 40 h according to time-lapse imaging. **d** Distribution of tracking duration for cells within the imaging area. **e** Distribution of travel distance for cells detected within the field of view over 40 h

Our transcriptomic analysis indicated that symbionts *in hospite* experience stronger nitrogen limitation than our initial nitrogen-limited cultures. To further examine the effects of nitrogen limitation observed *in hospite*, and to verify its effect on symbiont proliferation and life history regulation, we grew *S. microadriaticum* cells in a series of nitrogen-limited conditions (Fig. 5a; Additional file 2: Table S3). We found that total cell population and coccoid cell proportion were both inversely correlated with nitrogen concentration. Reduced nitrogen availability suppressed the proliferation of *S. microadriaticum* cells while up to 92.03% ± 0.63% (mean percentage ± s.e.m.) of the cells remained in the coccoid life stage (Fig. 5a). To figure out the potential confounding effects of total cell population and nitrogen level on coccoid proportion, we fitted these variables into a generalized linear model with coccoid proportion as the dependent variable. We found that both nitrogen level and total cell population played significant roles in determining coccoid proportions, but nitrogen level showed stronger negative effect (*t* = − 5.09, *p* = 0.0003) than the total cell population (*t* = − 2.41, *p* = 0.03).

Since all our analyses and experiments identified nitrogen as the limiting factor for cells *in hospite*, we further tested this hypothesis by tracking symbiont cell density changes in the coral *S. pistillata* supplied with different nutrients (Fig. 5b). It would be expected that the symbiont cell density increases only when the coral fragments were supplied with a nutrient that was limited *in hospite*. In line with our analysis, we found that there were no significant differences between corals supplied with iron (in the form of FeCl_3_) or phosphate (in the form of NaH_2_PO_4_) and the samples incubated in control conditions (normal seawater). Nitrogen supplement, however, increased cell density significantly, while ammonium showed a greater effect compared with nitrate (Fig. 5b). The different effects between ammonium and nitrate further proved that ammonium is the preferred nitrogen source of symbionts in corals [51, 52].

To further examine the effect of nitrogen limitation on symbiont cell life history, we tracked coccoid cells settled on a glass-bottom dish for over 40 h (Additional files 8 and 9: Videos S1, S2). Cells in the control condition showed significantly more detachment events (Additional file 8: Video S1), reflected in a drop in coccoid cell number at the beginning of the light period (Fig. 5c), followed by an increase after the onset of the dark period (Fig. 5c). This aligns well with the reported diel patterns of cell division and motility [53, 54]. However, no such pattern was observed for cells grown under nitrogen-limited conditions (Fig. 5c, Additional file 9: Video S2). Analysis of tracking durations and travel distances for cells within the field of view further revealed that nitrogen-limited cells perform differently from those in control conditions (Fig. 5d, e). Nitrogen-limited cells tended to stay on the glass bottom significantly longer, in prolonged tracking duration (Fig. 5d) and shorter travel distance (Fig. 5e). To further categorize different life stages, cells traveling extremely short distances, i.e., less than 10 μm (roughly the diameter of a single cell), were considered coccoids. Accordingly, 37.8% of the nitrogen-limited cells but only 4.0% of the cells in the control stay at the coccoid stage over the entire experimental period. The ratio of dividing cells (cell number increased) to motile cells (mastigotes) is significantly higher under nitrogen limitation than those in the control condition. These results indicated that nitrogen limitation plays a crucial role in cell life history control which is a key requisite for symbionts living inside the coral host cells.

## Discussion

Regulating the availability of critical nutrients has been suggested as the primary strategy for cnidarian hosts to control their symbionts [55, 56]. In our search for the potential mechanism, we performed parallel comparisons among all the essential nutrients affecting *S. microadriaticum* growth and found that nitrogen limitation could suppress the proliferation of *ex hospite* cells and induce a coccoid-dominant life cycle. Both of these phenotypes align with the features of symbionts *in hospite*. This indicates that nitrogen limitation is not only the means of symbiont population control but also plays essential roles in life history regulation for cells *in hospite*.

The importance of nitrogen in this photosynthetic symbiosis has been discussed from the host’s perspective [8, 10, 16], as well as from the symbiont side recently [41]. However, we present novel evidence of nitrogen limitation for symbiotic algae at the whole transcriptomic levels. While confirming the role of nitrogen *in hospite*, our study does not deny the importance of phosphate and iron for algal growth. Many important genes were affected once either of these elements were depleted (Additional file 5: Table S6, Additional file 6: Table S7). Fundamental processes, such as cell growth and photosynthesis, were suppressed when iron or phosphate was limited (Additional file 7: Table S8). However, due to the low similarity in gene expression profiles, coral symbionts do not seem to live in an environment restricted in phosphate or iron availability. This hypothesis was further supported by stable symbiont densities of coral fragments in nutrient-replete conditions where supplies of iron or phosphate did not promote symbiont cell proliferation. Our observations are, on the surface, contradictory to published observations reporting increases in symbiont cell densities in response to iron or phosphate supplementation [25, 33]. In our experiments, iron and phosphate supplementation did produce higher median symbiont cell densities than control, but the high number of replicates (*n* = 12) and large inter-replicate variances in cell densities rendered the increases insignificant (Fig. 5b). Our subsequent focus on nitrogen limitation in understanding the potential mechanisms underlying symbiont proliferation and life history in its coral host is driven by the extreme similarity between gene expression profiles of nitrogen-deplete and *in hospite* conditions.

Our spotlight on nitrogen is further supported by the observation that ammonium transporters and nitrate transporters were significantly induced by nitrogen limitation, both *in hospite* and *ex hospite* (Additional file 3: Table S4, Additional file 4: Table S5). This aligns well with previous studies on the green algae *Chlamydomonas reinhardtii* [57], the diatom *Phaeodactylum tricornutum* [58, 59], and the algal symbiont *Cladocopium goreaui* [60] and *Breviolum minutum* [41]. Our transcriptomic profiling allowed for a deeper investigation into the effects of nitrogen limitation and the response of symbionts competing within sub-optimal growth conditions. Nitrogen-depleted cells appear to upregulate a set of ammonium-producing biological processes while downregulating the nitrogen-consuming pathways. Purine metabolism, cyanoamino acid metabolism, and glycosaminoglycan degradation pathways would generate ammonium as one of their side products. We found these pathways were significantly upregulated under nitrogen-limited conditions. According to a recent hypothesis, *B. minutum* could scavenge nitrogen from purine when nitrogen is limited [41]. Additionally, our findings suggest that not only purine but also cyanoamino acid and glycosaminoglycan may serve as nitrogen sources when nitrogen is needed. In the meantime, pyrimidine metabolism and N-glycan biosynthesis were generally deactivated to avoid nitrogen consumption. Suppressing N-glycan biosynthesis, especially high-mannose type N-glycan biosynthesis, may play a more important role in establishing and maintaining this symbiotic relationship [61]. Enrichment of high-mannose N-glycan significantly decreases the colonization of *B. minutum* in *E. pallida* [61]. Hence, N-glycan profiles for algal cells in control and nitrogen-limited conditions could potentially be used as an indicator of suitable symbiont candidates.

Aside from these metabolic process changes, we report, for the first time, that nitrogen limitation alters the symbiont life history significantly. The pathways associated with flagella formation that are required for cell movement were significantly repressed. By contrast, the genes encoding cell adhesion molecules were upregulated. These changes are in line with the substantial increase of coccoid cells under nitrogen limiting conditions. Hence, we propose that nitrogen limitation is the main factor driving symbiont life history changes from mastigotes to coccoids. Motility is crucial for photosynthetic dinoflagellates as it enables vertical migration through the water column to maximize photosynthetic production and nutrient uptake [62]. These movements generally refer to the upward phototaxis for higher light intensity and downward chemotaxis for higher nitrogen concentration. The cost to maintain a flagellum is always high, especially for single-cell microorganisms [63], while the benefits strongly depend on nutrient availability. Flagella are high-nitrogen cellular organelles that contain many proteins, including actin, tubulin, cytoskeleton-associated motor proteins, and a diverse set of plasma membrane proteins on the flagellar surface [64, 65]. The cost of nitrogen for making these components could be well recovered by the benefits obtained from flagellar chemotaxis when nitrogen levels are high in the surrounding environment [62]. However, when nitrogen is scarce, the benefits from producing and operating such a high-nitrogen organelle might not return the costs of investments as mobility might still increase photosynthetic production but without nitrogen, the photosynthates produced cannot be used for proliferation. This cost-benefit imbalance may be the mechanism underlying the life history changes induced by nitrogen limitation. In our analyses looking into the potential connections between life history changes and reduced cell proliferation, we identified nine polycystin-2 that may be involved in both processes. Polycystin-2 is a calcium channel protein and highly conserved across metazoans [66]. In humans, polycystin-2 serves as a key regulator of the translation machinery that negatively controls human cell proliferation [67]. A homolog encoding for multiple polycystin-2 isoforms of varying lengths has also been identified in the green algae *Chlamydomonas reinhardtii* [68, 69]. Immunoblots indicated that different polycystin-2 isoforms are associated with vegetative or gametic *C. reinhardtii* cells, respectively, with only the shorter forms being found in flagella membranes. This may suggest that different forms of polycystin-2 play different roles in flagellum-based motility in *C. reinhardtii* [68]. The upregulation of polycystin-2 genes in *S. microadriaticum in hospite* and under nitrogen limitation suggests that it might serve as a pivotal regulator of both life history and cell proliferation processes.

Besides the above co-directional changes, symbionts *in hospite* induced specific nutrient-scavenging processes and unique photosynthetic system components. Endocytosis and downstream lysosomal pathways were generally suppressed in symbiotic cells. This indicates that cells *in hospite* do not rely on engulfing nutrients from the surrounding environment (symbiosome in this case). Nutrient limitation would inhibit photosynthesis efficiency in free-living algae [70]. However, symbiotic algae exhibited photosynthetic activities that are comparable with nitrogen-replete free-living samples [41]. There was no potential explanation for this phenomenon in previous studies. We propose that symbionts change components of their photosynthetic system *in hospite* by altering the expression of genes involved in carotenoid and antenna protein biosynthesis as well as circadian rhythm regulating molecules. These alterations may not only equip symbionts to better adapt to the light environment affected by auto-fluorescent proteins in coral hosts [71], but also to regulate their circadian clocks interactively with the host for optimal metabolic performance *in hospite* [72, 73]. However, more focused studies with tight control of light conditions for both symbionts in culture and *in hospite* are needed to examine this potential hypothesis.

We recognize that the nutrient concentrations in f/2 medium are 3–4 orders of magnitude higher than in natural conditions, and this may confound our interpretation of the results, especially in comparisons involving *in hospite S. microadriaticum* [74]. However, lab culture conditions often have a degree of artificiality in it for practical reasons—in particular, a rich medium is needed to obtain sufficient material for experimentation. Another concern is that our nutrient-limiting conditions are still “rich,” relative to natural conditions. One must also consider that the concentrations of our cultures are also multiple orders higher than natural conditions. On top of that, our nutrient limitations did achieve densities half that of control at stationary phase, and the long-term design (2 years) allowed the cultures sufficient time to adapt. We also strove to support our bioinformatics analyses with subsequent experimentation, e.g., by assessing the growth of algal cells, both in culture and *in hospite*, at different levels of nutrient limitation. We suggest future work in other strains should use cell densities (half that of control) as the comparative yardstick, instead of the nutrient levels we used to achieve that effect.

## Conclusions

In summary, our study show that nitrogen is the limiting factor for symbionts growing *in hospite* under natural conditions. Nitrogen limitation plays important roles not only in cell proliferation as previously hypothesized, but also in symbiont life history regulation. Our findings pave the way for a better understanding of the metabolic interactions between the coral host and its algal symbionts, while also providing novel directions for future research in functional biology of coral symbiosis.

## Methods

### Symbiodiniaceae cells and essential-nutrient screening

*Ex hospite Symbiodinium microadriaticum* (CCMP2467) cells, originally isolated from the coral *Stylophora pistillata*, were grown in an f/2 medium [75] at 25 °C and 80 μmol photons m^−2^ s^−1^ light under a 12-h:12-h light:dark cycle. At the beginning of this project, the strain had been maintained in our lab for more than 3 years and subcultured every 3 weeks.

To identify the essential nutrients necessary for the growth of *S. microadriaticum*, we first prepared the individual components of the f/2 medium (recipe listed in Additional file 2: Table S2) to produce final solutions with varying levels of nutrients (combinations of these components are in Additional file 2: Table S3). Cells were then grown in a standard f/2 medium (all components of the medium present) or in a modified f/2 medium with one specific component missing (i.e., complete depletion of either nitrate, phosphate, vitamins, iron, or other trace metals). As visible growth rate changes were detected only in cultures grown for over 6 months in nitrate-, phosphate-, or iron-depleted medium, we focused further efforts on reducing these three nutrients to concentrations that measurably limited cell proliferation.

Through trial-and-error, we modified the f/2 medium in three different ways: “1/4 N,” which contained nitrate at 1/4 of the concentration of a typical f/2 preparation; “1/8 P,” which contained phosphate at 1/8 strength; or “1/100 Fe,” which had iron at 1/100 strength (Additional file 2: Table S3). These nutrient levels were chosen because they reduced the growth of the cultures to approximately half of the control. These cultures, including controls (i.e., f/2 medium made from the same prepared components at the intended concentrations) were grown in triplicate. Afterward, the cells in these media were cultured for 2 years with subculturing every 3 weeks to acclimate the cells to nutrient-limited conditions. In the last subculture for this RNA-seq study, these cells were counted every 2 days using a Guava flow cytometer (Millipore) [76] starting from the sixth day after subculturing, and we harvested them at ~ 11:00 AM, 6 h into the light period of day 16 when they were at the late exponential phase. The cells were pelleted, snap-frozen in liquid N_2_, and temporarily stored at − 80 °C before RNA extraction.

### Counting of mastigote and coccoid in free-living cultures

To determine the effects of nutrient limitation on life history, we separated the motile mastigotes from the coccoid cells by carefully transferring the supernatant culture medium into a Falcon tube and subsequent centrifugation at 500*g* for 5 min and resuspension in 50 ml seawater. The remaining coccoid cells attached to the culture vessel were resuspended in 50 ml seawater; 10 μl of each of these cell suspensions was counted right after collection using a Countess II FL Automated Cell Counter (Thermo Fisher). The total numbers of mastigote and coccoid cells were calculated for each flask and used for percentage and ratio calculations. Paired *t*-tests were performed on the coccoid/mastigote ratios to evaluate the statistical significance of relative cell stage changes in response to nutrient depletions. Fisher’s exact tests were applied on mastigote and coccoid numbers to calculate the effects of nutrient limitation on cell life stage changes.

To examine the severity of nitrogen limitation *in hospite* and to mimic its effects on symbiont cell proliferation and life history regulation, we subcultured *S. microadriaticum*, directly from cells grown in full f/2 medium, under a series of nitrogen-reduced conditions: 440 μM, 220 μM, 110 μM, 55 μM, and 27.5 μM, representing 1/2, 1/4, 1/8, 1/16, and 1/32 of the nitrogen level in standard f/2 medium, respectively (Additional file 2: Table S3). The total cell populations and the corresponding coccoid proportions were determined at 11 AM, 6 h into the light period, on the 15th day post-subculturing.

### RNA extraction and sequencing

To explore the gene expression changes in response to different nutrient conditions, total RNA was extracted as described previously [77]. Briefly, *S. microadriaticum* cells collected from 300 ml liquid cultures were ground in pre-chilled mortars and pestles together with 50 μl of 0.5-mm-diameter glass beads. Six hundred microliters of Qiagen RLT buffer and 10 μl of 2-mercaptoethanol were added to aid cell lysis and to prevent RNA degradation. The RNA was then isolated using the RNeasy Mini Kit (Qiagen) by following the protocol for extraction from animal tissues in the manufacturer’s manual. The purified RNA was quantified using a Qubit 2.0 (Thermo Fisher), and quality-checked on a Bioanalyzer (Agilent Technologies) using an Agilent RNA 6000 Nano Kit. All RNA samples showed a RIN value greater than 7 that indicated good RNA integrity suitable for downstream transcriptomic profiling.

Sequencing libraries were constructed from oligo-dT selected mRNA using an Illumina TruSeq RNA Library Preparation Kit v2 according to the manufacturer’s instructions. A total of 390 million 150-bp paired-end reads were retrieved from 2 lanes on the Illumina HiSeq 4000 platform for 12 *S. microadriaticum* samples. All the raw sequencing reads were deposited in the Sequence Read Archive under BioProjects PRJNA613780 and PRJNA386774.

### Differential expression analysis

To compare the transcriptomic profiles of *ex hospite S. microadriaticum* and *in hospite* symbionts, we included RNA-seq data from the coral *Stylophora pistillata*, the original host of *S. microadriaticum*. The coral samples were fully symbiotic and their RNA-seq data contained significant amounts of sequenced reads originating from their symbiont *S. microadriaticum* [78]. To further confirm the symbiont species in both datasets, we first mapped the RNA-seq data against the SymPortal reference database that contains thousands of ITS2 sequence data from all Symbiodiniaceae genera [79] using kallisto v0.44.0 [80]. Based on this analysis, we confirmed that the vast majority of the reads (> 98 %) in all datasets originated from *S. microadriaticum* (formerly clade A1).

The genomic gene models of *S. microadriaticum* [81] were then used for all subsequent quantification and differential expression analyses. The reads were trimmed using TrimGalore v0.4.5 [82] with default parameters to remove adapter sequences and the low-quality bases. Gene expression levels of *S. microadriaticum* from the different conditions were quantified and normalized using kallisto v0.44.0 [80]. Differential gene expression analyses were done for the comparisons between control and each of the rest conditions using sleuth v0.29.0 [83]. A gene was identified as differentially expressed when its *q*-value (multiple test corrected *p*-value) was less than 0.05.

### Analysis of differential pathway activities

Pathway analyses were performed on the 260 KEGG pathways annotated based on the *S. microadriaticum* genome [81] using gene set variation analysis (GSVA), a non-parametric unsupervised gene set enrichment method implemented in GSVA R package [84]. The gene sets were simplified as previously reported [50]. Briefly, to reduce gene redundancy, each pathway-associated gene set was trimmed with only unique genes. All the genes associated with more than one pathway were removed to avoid pathway overlaps. After the cleaning, 159 pathways were retained for GSVA and downstream differential activity analysis.

Pathway activity as represented by GSVA scores was clustered and visualized using ComplexHeatmap [85]. The output of GSVA is a pathway by sample matrix of GSVA scores that are normally distributed. To estimate the differential activities of pathways between different conditions, we contrasted the activity scores from each of the nutrient-limited conditions and the symbiotic samples with the scores for control free-living cells using a generalized linear model implemented in limma [49].

### Gene Set Enrichment Analysis (GSEA)

*S. microadriaticum* genome-annotated Gene Ontology (GO) terms were taken as reference gene sets. The enrichment score and its significance level were calculated for each of these gene sets using GSEA [86]. This was done on DEGs identified in each of the differential expression analyses, using the *GSEA* function implemented in clusterProfiler [87]. A gene set was considered enriched only when its adjusted *p*-value was less than 0.05.

### Determination of symbiont cell density in corals at nutrient-replete conditions

To test the effect of nutrient repletion on cell proliferation of symbionts *in hospite*, we incubated fragments of the coral *S. pistillata* in autoclaved seawater enriched with phosphate (NaH_2_PO_4_), iron (FeCl_3_), ammonium (NH_4_Cl), or nitrate (NaNO_3_).

The coral colonies were collected in the central Red Sea (Al Fahal Reef, 22° 14′ 54″ N 38° 57′ 46″ E) and acclimatized in indoor tanks with constant sediment-filtered Red Sea water in-flow (salinity ~ 39–40 ppt) for at least 3 months. Twelve coral branches from different colonies were cut for each of the four nutrient treatments and the ambient seawater control, then tied to plastic stands and placed into three transparent Nalgene™ straight-sided wide-mouth polycarbonate jars (Thermo Fisher). Two hundred fifty milliliters of seawater from indoor acclimation tanks was used to fill up each of the jars, and the water was changed every 2 days. To ensure sufficient oxygen levels in such small volumes, we added magnetic stirring bars to the jars before placing them onto a Cimarec™ i Telesystem Multipoint Stirrer (Thermo Fisher) with constant stirring at 300 rpm. The whole setup was placed in an incubator at 25 °C with ~ 80 μmol photons m^−2^ s^−1^ radiation and a 12-h light/12-h dark cycle (Additional file 1: Fig. S4). Coral fragments were acclimated in ambient seawater for 2 days before nutrient-repletion treatments.

The starting concentrations of nitrogen, phosphate, and iron were calculated based on their ratios in the f/2 medium that was used for free-living *S. microadriaticum* culture. We then adjusted the nutrients, following the ratios, to the levels that did not cause visible stress effects on corals (i.e., the corals grew with no observable defects in the treatments over 2 weeks): ammonium 250 μM, nitrate 250 μM, phosphate 10.2 μM, or iron 3.316 μM.

Coral fragments were collected after 6 days of nutrient-replete incubation and airbrushed with a lysis buffer (0.2 M Tris-HCl, pH = 7.5; 0.5% Triton-X; 2 M NaCl) to collect the animal tissue. The tissue lysates were sheared by repeated passage through a 25-G needle to release the symbionts; 500 μL of each homogenized sample was centrifuged to pellet symbionts for cell counting and to separate supernatants to determine host protein content. Symbiont cell pellets were resuspended, and cells were counted based on their chlorophyll fluorescence using a BD LSRFortessa™ Cell Analyzer (BD Biosciences). Host protein content was measured with a Pierce Micro BCA™ Protein Assay Kit (Thermo Fisher) according to the manufacturer’s recommendations. Cell density was determined by normalizing the total cell number to coral protein content. The normality of cell density data was tested using the Shapiro-Wilk test and followed by Levene’s test of homogeneity of variance. Statistical differences among conditions were calculated using one-way ANOVA with Games-Howell post hoc tests.

### Observation of cell proliferation using time-lapse imaging

To examine the effect of nitrogen limitation on symbiont life history, we performed a time-lapse experiment to track coccoid cells attached to glass-bottom dishes for ~ 40 h, with images taken every 5 min, using Leica TCS SP8 STED microscope (Leica). A total of 490 images were captured and converted into a video at a frame rate of 21 frames per second for each of the two conditions, control and nitrogen depletion. The number, travel distance, and tracking duration of cells for each condition were analyzed using Fiji [88] and the TrackMate plugin [89].

## Supplementary Information

**Additional file 1: Fig. S1.** Overview of the experimental workflow. **Fig. S2.** Overlaps between differentially expressed genes identified from comparisons of Spis vs Con, N vs Con, Fe vs Con, and P vs Con. **Fig. S3.** Spearman correlation of expression changes between *in hospite* and iron- (A), phosphate- (B), or nitrogen-limited *S. microadriaticum* (C), in comparison with control cells, respectively. **Fig. S4.** Setup for small-scale short-term coral culturing in a laboratory environment.

**Additional file 2: Table S1.** Symbiotic cnidarian species searched for SLL-2 homologs. **Table S2.** Chemical composition of stock solutions in f/2 medium. **Table S3.** Set-up of nutrient stress subcultures.

**Additional file 3: Table S4.** Differentially expressed genes identified from the comparison between cells in hospite and in f/2 medium.

**Additional file 4: Table S5.** Differentially expressed genes identified from the comparison between cells in nitrogen-depleted and standard f/2 medium.

**Additional file 5: Table S6.** Differentially expressed genes identified from the comparison between cells in phosphate-depleted and standard f/2 medium.

**Additional file 6: Table S7.** Differentially expressed genes identified from the comparison between cells in iron-depleted and standard f/2 medium.

**Additional file 7: Table S8.** KEGG pathway activity analysis.

**Additional file 8: Video S1.** Time-lapse imaging of algal cells in control condition.

**Additional file 9: Video S2.** Time-lapse imaging of algal cells in nitrogen-limited condition.

## Data Availability

All the raw sequencing reads were deposited in the Sequence Read Archive under BioProjects PRJNA613780 [90] and PRJNA386774 [91].

## Abbreviations

DEGs: Differentially expressed genes
GSVA: Gene set variation analysis
GSEA: Gene set enrichment analysis
GO: Gene Ontology
