# Supplementary material for: Nutritional control regulates symbiont proliferation and life history in coral-dinoflagellate symbiosis
Source: BMC Biol. 2022 May 13;20:103. doi: 10.1186/s12915-022-01306-2 (PMC9102920; doi:10.1186/s12915-022-01306-2)
Supplement: Supplementary file 1 — Additional file 1: Fig. S1. Overview of the experimental workflow. Fig. S2. Overlaps between differentially expressed genes identified from comparisons of Spis vs Con, N vs Con, Fe vs Con, and P vs Con. Fig. S3. Spearman correlation of expression changes between in hospite and iron- (A), phosphate- (B), or nitrogen-limited S. microadriaticum (C), in comparison with control cells, respectively. Fig. S4. Setup for small-scale short-term coral culturing in a laboratory environment. [file 12915_2022_1306_MOESM1_ESM.pdf]

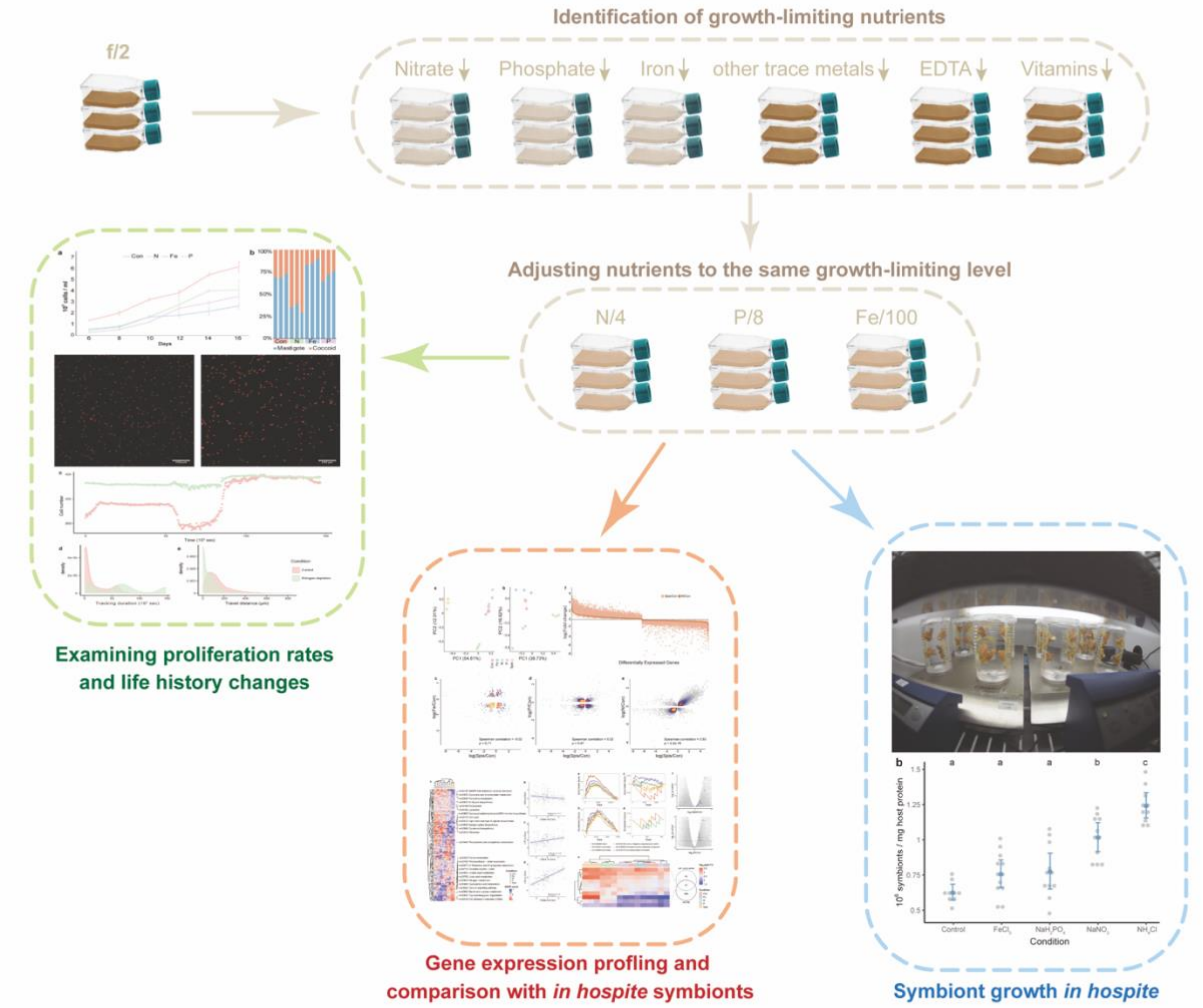

Fig. S1 Overview of the experimental workflow. Growth-limiting conditions were first determined based on the proliferation rates of free-living cells under different nutrient depletion conditions. Gene expression profiles of algal cultures in these nutrient-limited conditions were examined using RNA-Seq and compared with gene expression profiles from symbionts residing in their coral host *Stylophora pistillata*. The effects of nutrient depletions on cell proliferation and life history changes were tested in vitro using multiple strategies, including cell counting and time-lapse imaging. Final validation in hospite were performed by monitoring symbiont growth in S. pistillata nubbins subjected to different nutrient-replete conditions.

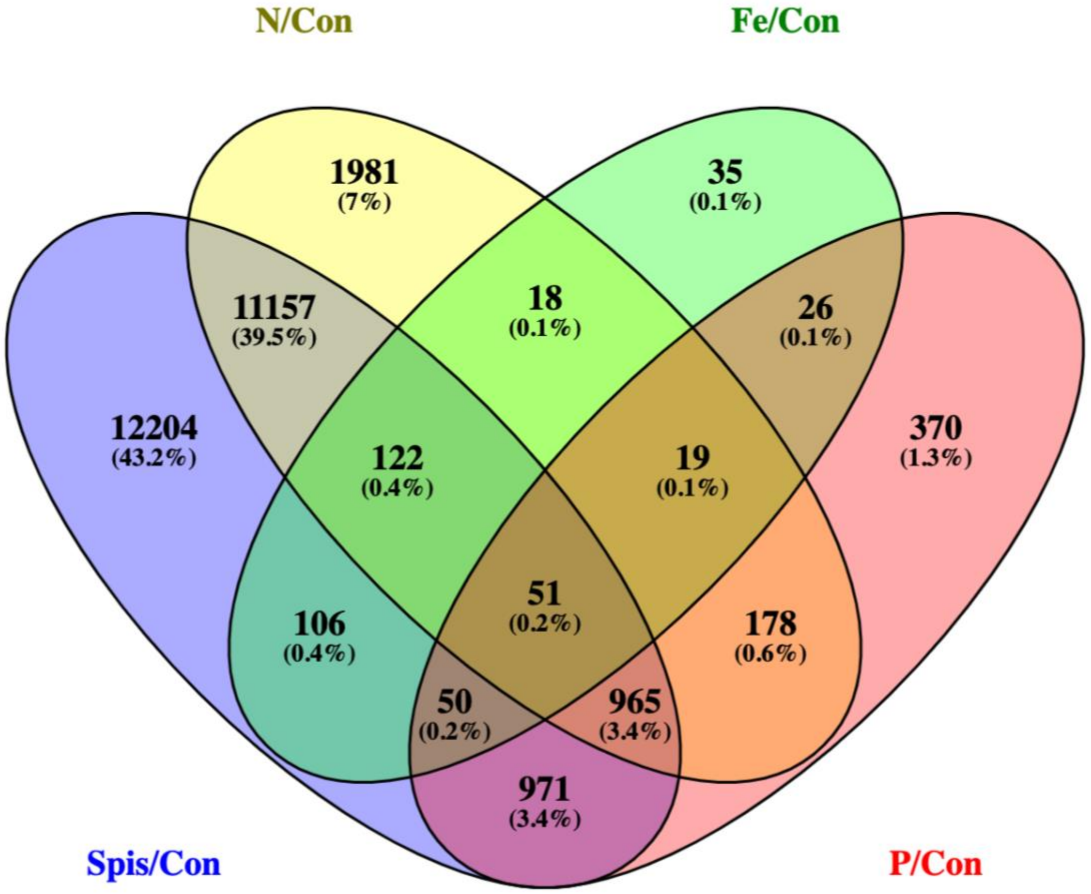

Fig. S2 Overlaps between differentially expressed genes identified from comparisons of Spis vs Con, N vs Con, Fe vs Con, and P vs Con.

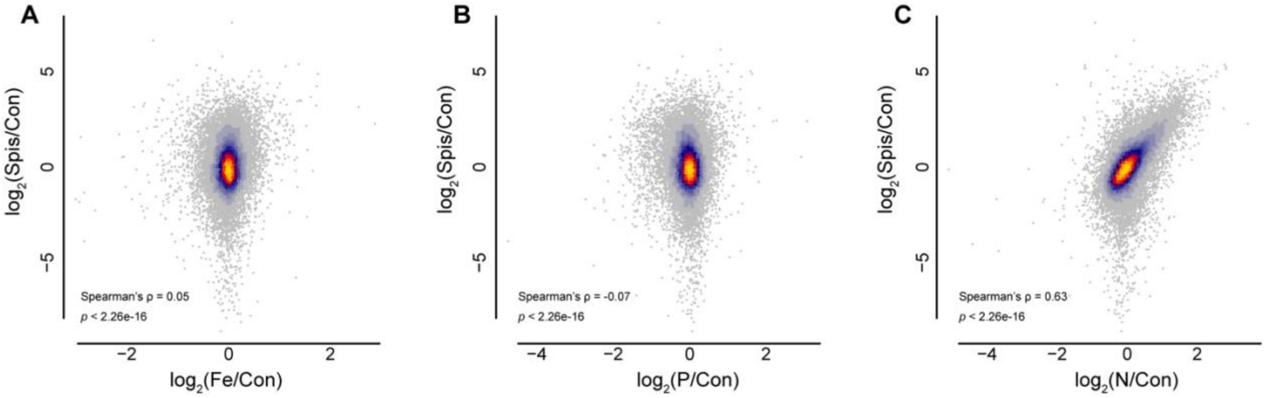

Fig. S3 Spearman correlation of expression changes between *in hospite* and iron- (**A**), phosphate- (**B**), or nitrogen-limited *S. microadriaticum* (**C**), in comparison with control cells, respectively. Each dot represents the expression change of a gene expressed at both conditions in comparison with *ex hospite* control. The correlation coefficient and *p*-value were calculated based on their log-transformed fold-changes. Colors represent the dot density which corresponds to increasing density from gray, blue, red, to yellow.

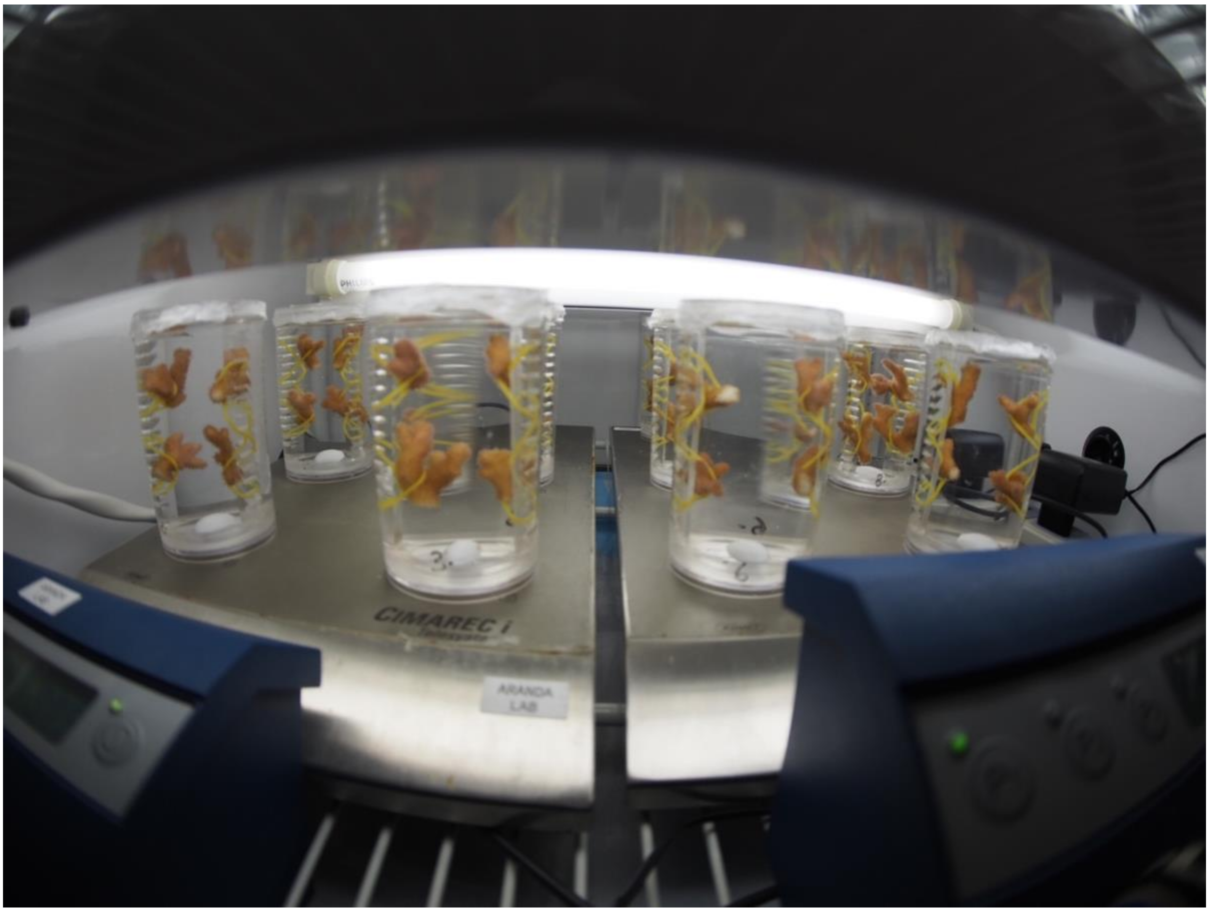

Fig. S4 Setup for small-scale short-term coral culturing in a laboratory environment. Coral branches were tied on plastic stands, and placed into three transparent Nalgene™ straight-sided wide-mouth polycarbonate jars (2116-0250, Thermo Fisher Scientific, Waltham, MA). 250 ml seawater used to fill up each of the jars was changed every two days. To ensure efficient oxygen levels in such small volumes, jars with magnetic stirring bars were placed onto Cimarec™ i Telesystem Multipoint Stirrers (50088034, Thermo Fisher Scientific, Waltham, MA) and stirred at 300 rpm constantly. The whole setup was placed into an incubator at 25°C with ~40 µmol photons $m^{-2}s^{-1}$ radiation and a 12-hour light/12-hour dark cycle.
