# Supplementary material for: Nutritional control regulates symbiont proliferation and life history in coral-dinoflagellate symbiosis
Source: BMC Biol. 2022 May 13;20:103. doi: 10.1186/s12915-022-01306-2 (PMC9102920; doi:10.1186/s12915-022-01306-2)
Supplement: Supplementary file 2 — Additional file 2: Table S1. Symbiotic cnidarian species searched for SLL-2 homologs. Table S2. Chemical composition of stock solutions in f/2 medium. Table S3. Set-up of nutrient stress subcultures. [file 12915_2022_1306_MOESM2_ESM.pdf]

Table S1 Symbiotic cnidarian species searched for SLL-2 homologs

	Species	References
**Reef-building corals**	*Acropora digitifera*	Shinzato et al. (2011)
	Acropora tenuis	Voolstra et al. (2015)
	Fungia spp.	Voolstra et al. (2015)
	Galaxea fascicularis	Voolstra et al. (2015)
	Goniastrea aspera	Voolstra et al. (2015)
	Pocillopora damicornis	Cunning et al. (2018)
	Pocillopora verrucose	Buitrago-Lopez et al. (2020)
	Porites lutea	Voolstra et al. (2015)
	Stylophora pistillata	Voolstra et al. (2017)
	Pachyseris speciosa	Voolstra et al. (2015)
**Corallimorpharia**	*Amplexidiscus fenestrafer*	Wang et al. (2017)
	Discosoma sp.	Wang et al. (2017)
**Sea anemones**	*Actinia equina*	Wilding et al. (2020)
	Actinia tenebrosa	van der Burg et al. (2016)
	Aiptasia pallida	Baumgarten et al. (2015)
	Anthopleura buddemeieri	van der Burg et al. (2016)
	Aulactinia veratra	van der Burg et al. (2016)
	Calliactis polypus	van der Burg et al. (2016)
	Nemanthus annamensis	van der Burg et al. (2016)
	*Telmatactis sp.*C	van der Burg et al. (2016)

Baumgarten, S., Simakov, O., Esherick, L.Y., Liew, Y.J., Lehnert, E.M., Michell, C.T., Li, Y., Hambleton, E.A., Guse, A., Oates, M.E., *et al.* (2015). The genome of Aiptasia, a sea anemone model for coral symbiosis. Proc Natl Acad Sci U S A *112*, 11893-11898.

Buitrago-Lopez, C., Mariappan, K.G., Cardenas, A., Gegner, H.M., and Voolstra, C.R. (2020). The Genome of the Cauliflower Coral Pocillopora verrucosa. Genome Biol Evol *12*, 1911-1917.

Cunning, R., Bay, R.A., Gillette, P., Baker, A.C., and Traylor-Knowles, N. (2018). Comparative analysis of the Pocillopora damicornis genome highlights role of immune system in coral evolution. Sci Rep *8*, 16134.

Shinzato, C., Shoguchi, E., Kawashima, T., Hamada, M., Hisata, K., Tanaka, M., Fujie, M., Fujiwara, M., Koyanagi, R., Ikuta, T., *et al.* (2011). Using the Acropora digitifera genome to understand coral responses to environmental change. Nature *476*, 320-323.

van der Burg, C.A., Prentis, P.J., Surm, J.M., and Pavasovic, A. (2016). Insights into the innate immunome of actiniarians using a comparative genomic approach. Bmc Genomics *17*.

Voolstra, C.R., Li, Y., Liew, Y.J., Baumgarten, S., Zoccola, D., Flot, J.F., Tambutte, S., Allemand, D., and Aranda, M. (2017). Comparative analysis of the genomes of Stylophora pistillata and Acropora digitifera provides evidence for extensive differences between species of corals. Sci Rep *7*, 17583.

Voolstra, C.R., Miller, D.J., Ragan, M.A., Hoffmann, A.A., Hoegh-Guldberg, O., Bourne, D.G., Ball, E.E., Ying, H., Foret, S., Takahashi, S., *et al.* (2015). The ReFuGe 2020 Consortium-using "omics" approaches to explore the adaptability and resilience of coral holobionts to environmental change. Front Mar Sci *2*.

Wang, X., Liew, Y.J., Li, Y., Zoccola, D., Tambutte, S., and Aranda, M. (2017). Draft genomes of the corallimorpharians Amplexidiscus fenestrafer and Discosoma sp. Mol Ecol Resour *17*, e187-e195.

Wilding, C.S., Fletcher, N., Smith, E.K., Prentis, P., Weedall, G.D., and Stewart, Z. (2020). The genome of the sea anemone Actinia equina (L.): Meiotic toolkit genes and the question of sexual reproduction. Mar Genomics *53*, 100753.

Table S2 Chemical composition of stock solutions in f/2 medium

Chemicals	Concentrations
**1. Nitrate stock (1,000 x)**	
NaNO3	75 g/L
**2. Na2EDTA stock (1,000 x)**	
$Na_2EDTA \cdot 2H2O$	4.36 g/L
**3. Trace metals stock (10,000 x)**	
$CuSO_4 \cdot 5H_2O$	100 mg/L
$ZnSO_4 \cdot 7H_2O$	220 mg/L
$CoCl_2 \cdot 6H_2O$	100 mg/L
$MnCl_2 \cdot 4H_2O$	1.8 g/L
$Na_2MoO_4 \cdot 2H_2O$	60 mg/L
**4. Iron stock (1,000 x)**	
$FeCl_3 \cdot 6H_2O$	3.15 g/L
**5. Phosphate stock (1,000 x)**	
NaH_2PO_4	4.411 g/L
**6. Vitamins stock (100,000 x)**	
Biotin	500 mg/L
Vitamin	B12 500 mg/L
Thiamine HCl	10 g/L

Table S3 Set-up of nutrient stress subcultures

	Control (f/2)	1/2 N	1/4 N	1/8 N	1/16 N	1/32 N	1/100 Fe	1/8 P
**Previous culture**	1 ml	1 ml	1 ml	1 ml	1 ml	1 ml	1 ml	1 ml
**Autoclaved seawater**	100 ml	100 ml	100 ml	100 ml	100 ml	100 ml	100 ml	100 ml
**Stock solutions (from Table S1)**								
**1: Nitrate**	100 µl	50 µl	25 µl	12.5 µl	6.25 µl	3.13 µl	100 µl	100 µl
**2: EDTA**	10 µl	10 µl	10 µl	10 µl	10 µl	10 µl	10 µl	10 µl
**3: Trace metals**	100 µl	100 µl	100 µl	100 µl	100 µl	100 µl	100 µl	100 µl
**4: Iron**	100 µl	100 µl	100 µl	100 µl	100 µl	100 µl	1 µl	100 µl
**5: Phosphate**	100 µl	100 µl	100 µl	100 µl	100 µl	100 µl	100 µl	12.5 µl
**6: Vitamins**	1 µl	1 µl	1 µl	1 µl	1 µl	1 µl	1 µl	1 µl
